# Migraine is *first* cause of disability in under 50s: will health politicians now take notice?

**DOI:** 10.1186/s10194-018-0846-2

**Published:** 2018-02-21

**Authors:** Timothy J. Steiner, Lars J. Stovner, Theo Vos, R. Jensen, Z. Katsarava

**Affiliations:** 1 Department of Neuromedicine and Movement Science, NTNU Norwegian University of Science and Technology, Edvard Griegs Gate, Trondheim, Norway; 2 Division of Brain Sciences, Imperial College London, London, UK; 3 Norwegian Advisory Unit on Headache, Department of Neurology and Clinical Neurophysiology, St Olavs University Hospital, Trondheim, Norway; 4 Institute for Health Metrics and Evaluation (IHME), University of Washington, Seattle, WA USA; 5 Danish Headache Centre, Department of Neurology, University of Copenhagen, Rigshospitalet Glostrup, Glostrup, Denmark; 6 Department of Neurology, Evangelical Hospital Unna, Unna, Germany; 7 Medical Faculty, University of Duisburg-Essen, Essen, Germany

**Keywords:** Headache disorders, Migraine, Tension-type headache, Medication-overuse headache, Burden of disease, Disability, Public health, Global Burden of Disease study, Global Campaign against Headache

If it were needed, more evidence of the disconcerting under-treatment of headache disorders has come from the Eurolight study [1]. The topic is not new. Twenty years ago, the International and American Headache Societies jointly voiced their dismay at the inadequacies of health care for headache [2]. In 2006, the European Headache Federation and World Headache Alliance described migraine as a “forgotten epidemic” [3]. Meanwhile, in 2003, the Global Campaign against Headache [4–6] engaged the World Health Organization (WHO) as partner in this cause [7], embarking on a worldwide action programme which began by assessing the magnitude of headache in the world [4, 8]. In 2011, WHO’s global survey of headache disorders and resources, a Global Campaign project, laid bare the scale and scope of under-treated headache everywhere, and its consequences [9]. WHO wrote, in a message sent inter alia to the world’s Ministries of Health: “This first global enquiry into these matters illuminates the worldwide neglect of a major public-health problem, and reveals the inadequacies of responses to it in countries throughout the world” [9]. No words could be clearer but, to make sure, WHO repeated the message soon after [10].

Eurolight was a cross-sectional survey of over 8000 participants, conducted by multiple partners (scientific and lay) in 10 European countries [11]. A considerable strength of this study, apart from its size and geographical scope, was the use in all countries of the same questionnaire [12], a derivative of the HARDSHIP questionnaire already employed in many different countries, cultures and translations [13]. Also a strength was its scope of enquiry, simultaneously into migraine, tension-type headache (TTH) and medication-overuse headache (MOH), the three headache disorders of major public-health importance. This provided a broad view of headache in Europe. The different sampling methods employed by the countries in Eurolight produced samples that varied in their representativeness of the general population, arguably a strength in that it brought data into the survey from diversely-sourced samples [11, 12].

The new report analyses Eurolight data for indicators of adequacy of medical care [1]. The focus is on migraine, and the findings are depressing. Among 1175 participants in the 10 countries reporting frequent migraine – on more than five days per month, indicating unambiguous need for preventative medication – fewer than 20% had seen a health-care professional (general practitioner [GP] or specialist). In most countries, fewer than 10% were receiving what might be considered adequate acute treatment, and even smaller proportions had the preventative medication for which they were clearly eligible. Participants who had managed to make contact with specialists generally received better care by these indicators, which might be expected. Those seeing GPs were less well served, and those entirely dependent on self-medication – the large majority – fared poorly. In other words, the authors conclude, in wealthy Europe, too few people with migraine consult physicians, and migraine-specific medications are used inadequately even among those who do [1]. Is there hope at all for people with headache in less well-resourced countries?

The Eurolight report comes soon after publication of the latest (2016) Global Burden of Disease (GBD) study [14]. “The most comprehensive worldwide observational epidemiological study to date” [15], GBD has been performed reiteratively since 1990, with estimates of health loss due to disease a principal objective [16]. Its findings, informing national health policies, offer a rational basis for priority setting and resource-allocation, driving service organisation and delivery to meet assessed needs. GBD now revises its estimates annually as it continuously develops and refines the methodology of disease-burden estimation and its expression as premature mortality (years of life lost: YLLs) and disability (years lived with disability: YLDs). At the same time, updated estimates take account of new epidemiological evidence as it continues to become available.

Since migraine was first included in GBD, it has ascended the ranks of top causes of YLDs worldwide, from its debut at 19th in GBD 2000 [17] to seventh in GBD 2010 [18, 19] and sixth in GBD 2013 [20, 21]. This persistent rise is not indicative of increasing prevalence: it follows the collection and assimilation into GBD of ever better data as new population-based studies have slowly filled the large knowledge gaps, which as recently as 2007 related to more than half the world’s population [22]. With better knowledge, empirical data have replaced many of the assumptions underlying the earlier GBD estimates, and, as YLD calculations became prevalence-based rather than reliant on the less-easily ascertained incidence and duration, estimates have gained in reliability. In GBD 2015, migraine dropped back to seventh among causes of YLDs, partly because of revised estimates for other disorders, but, being notably age-related, it was third in both males and females aged 15–49 [23].

GBD 2016 offers sobering findings for those affected by or who care about migraine [14]. At level two of GBD’s disease hierarchy, neurological disorders collectively account for 8.6% of all YLDs in the world, and come fourth in the disability ranking (behind mental and substance use, “other non-communicable” and musculoskeletal disorders). At level three, headache disorders are the cause of *more than three quarters* of all neurological YLDs (6.5% of all YLDs), despite that neurological disorders include epilepsy, Alzheimer disease and other dementias, Parkinson’s disease, multiple sclerosis and motor neuron disease. At level four, migraine now takes *second place*, responsible for 5.6% of all YLDs in the world, behind only low back pain (7.2%) (Table 1).

**Table 1:** Top 10 level-4 causes of disability in GBD 2016 (global, both sexes, all ages)

Low back pain	
Migraine	
Age-related hearing loss	
Iron-deficiency anaemia	
Major depression	
Neck pain	
Other musculoskeletal disorders	
Diabetes	
Anxiety disorders	
Falls	

There is worse. In the age group 15–49 years, migraine is the *top* cause of YLDs [14] (Table 2). Let us not forget that these are the productive years, when education is completed, families formed, children raised, careers built and prospects for the whole remainder of life established. Whatever impact migraine-attributed disability may have more generally, during these years it is greatly magnified.

**Table 2:** GBD 2016: Years lived with disability (YLDs) attributed to migraine by gender, age and region (from [14])

Region	Gender	Age range (years)	% of total YLDs (95% CI)	Rank
Global	Both	All 15–49	5.6 (4.0–7.2) 8.2 (6.0–10.6)	2 1
	M	All 15–49	4.3 (3.1–5.5) 6.4 (4.6–8.2)	3 2
	F	All 15–49	6.8 (4.9–8.8) 9.8 (7.1–12.7)	2 1
North America	Both	All	4.8 (3.5–6.1)	5
Latin America and Caribbean			6.7 (4.9–8.6)	2
Western Europe			6.2 (4.5–7.9)	2
Central and Eastern Europe and Central Asia			6.0 (4.4–7.7)	3
South Asia			6.5 (4.6–8.5)	2
SE and East Asia and Oceania			4.6 (3.3–6.0)	4
North Africa and Middle East			6.7 (5.0–8.6)	2
Sub-Saharan Africa			4.6 (3.2–6.1)	3

There is a ready explanation for the apparently steep rise in migraine since GBD 2015, conducted a year earlier: it lies with MOH. GBD 2015 regarded MOH as a separate disease [23]. While MOH is relatively uncommon (prevalence estimates vary around the world but are mostly in the range 1.5–3% [24, 25]), it is highly disabling, by definition characterised by headache on 15 or more days per month [26]. GBD 2015 placed it 18th among the causes of YLDs [23]. Nosologically, MOH is undoubtedly a distinct disease [26], but aetiologically it is a complication arising from mistreatment of other headache disorders, principally migraine and to a lesser extent TTH: it does not occur otherwise [26]. In GBD 2016, the decision was made that burden attributed to MOH would be more correctly attributed to the antecedent disorders, in due proportion (73.4% to migraine, 26.6% to TTH, from a meta-analysis of three studies [27–29]).

Not everybody may agree with this, but there is both logic and purpose in recognising MOH as one of the *sequelae* (health states) of the antecedent headache disorder. In GBD terms, therefore, migraine is associated with three potential health states, each occurring with measurable probability (established in population-based studies): the ictal state (during an attack, with its symptoms), the interictal state (between recurrent attacks), and MOH. All three contribute to the disability burden of migraine, and all three contributions should be duly recognised. (We noted earlier that GBD does not consider disability associated with the interictal state of headache disorders [21], although significant interictal burden is reported by many people with migraine [30]).

From GBD 2016 it is more evident than ever that headache disorders have a very large detrimental effect on public health. Table 2 shows that migraine is a major contributor to disability throughout the world, in both high- and low-income countries [14]. It is worth noting that, of the 21 regions into which GBD divides the world, five are still without any data on headache and more have only scarce data. Furthermore, most data are from adults, with relatively few studies reporting on children and adolescents. Nevertheless, headache disorders are, manifestly, an egregious cause of health loss. Why, then, when efficacious and cost-effective treatments exist [31, 32], do health services almost everywhere leave them side-lined [9, 10, 33]? Will health politicians finally take notice, now that migraine is top of the heap?

Looking forward, and not to end on an impliedly negative note, we remind researchers that further population-based studies are needed to fill the remaining knowledge gaps. Quality in these is all-important: published methodological guidelines [34] and instruments [13] are available, and surveys should follow and adopt these. Ultimately, if studies contributing to GBD are standardized, future iterations of GBD may not only show the relative importance of headache in global public health but also monitor the benefits of improvements in headache care, new treatments and societal change.
